# Basophil Activation Test in IgE-Mediated Wheat Allergy: Diagnostic and Clinical Applications—A Narrative Review

**DOI:** 10.3390/diagnostics15202659

**Published:** 2025-10-21

**Authors:** Elena Camelia Berghea, Mădălina Coman-Stanemir, Ioana Raluca Papacocea

**Affiliations:** 1 Department of Paediatrics, “Carol Davila” University of Medicine and Pharmacy, 020021 Bucharest, Romania; camelia.berghea@umfcd.ro; 2 Department of Allergology and Clinical Immunology, “Marie S. Curie” Emergency Children’s Clinical Hospital, 077120 Bucharest, Romania; 3 Department of Medical Physiology, Faculty of Medicine, Carol Davila University of Medicine and Pharmacy, 020021 Bucharest, Romania

**Keywords:** basophil activation test, wheat allergy, IgE-mediated allergy, wheat-dependent exercise-induced anaphylaxis, component-resolved diagnostics, omega-5 gliadin, hydrolyzed wheat protein, anaphylaxis biomarkers, omalizumab, functional flow cytometry

## Abstract

The basophil activation test (BAT) is an emerging tool in the diagnosis and management of IgE-mediated wheat allergies (WAs), particularly in complex or high-risk phenotypes. This narrative review explores the clinical applications of BAT across a spectrum of WA presentations, including wheat-dependent exercise-induced anaphylaxis (WDEIA), contact urticaria, and pediatric food allergy. The BAT provides a functional measure of allergen-triggered basophil activation, bridging the gap between mere sensitization and true clinical reactivity. We highlight its utility in detecting sensitization to specific wheat components such as ω-5 gliadin, glutenin subunits, and hydrolyzed wheat proteins, and its value in cases where traditional diagnostics are inconclusive. Furthermore, BAT is discussed as a dynamic biomarker in therapeutic monitoring, especially in patients receiving omalizumab, where reduced basophil reactivity correlates with improved clinical outcomes. While standardization and access remain challenges, the BAT holds significant promise as a precision diagnostic and monitoring tool in wheat allergies.

## 1. Introduction

Wheat (Triticum spp.) is a globally consumed staple grain, playing a crucial role in human diets, but is also a well-recognized IgE-mediated food allergen worldwide [1]. Within the clinical spectrum of wheat allergies, patients may experience from only mild symptoms such as erythema, pruritus, or gastrointestinal discomfort to severe cases that can progress to systemic anaphylaxis. A notable and potentially life-threatening form is wheat-dependent exercise-induced anaphylaxis (WDEIA), a cofactor-dependent syndrome that is under-recognized and misdiagnosed (for example, as idiopathic anaphylaxis) unless specifically tested [2].

Recent epidemiological data indicate that wheat allergy is increasingly recognized across Asia, particularly in Japan, Korea, and China, where dietary westernization has expanded wheat consumption. This emerging trend contrasts with Europe, where wheat allergy has long been documented and characterized by well-defined phenotypes. The regional variation likely reflects differences in dietary exposure, genetic background, and allergen component sensitization profiles [3,4].

Diagnosing IgE-mediated wheat allergy is challenging. Wheat proteins are complex, encompassing multiple allergenic components (including water-/salt-soluble albumins/globulins and alcohol-soluble gliadins and glutenins). Conventional tests have limitations: skin-prick testing and wheat-specific IgE assays are sensitive but lack specificity. Oral food challenge (OFC) remains the definitive “gold-standard” diagnostic test for IgE-mediated wheat allergy [5]. The basophil activation test offers a functional measure of allergen reactivity that complements existing diagnostics. Current studies in pediatric and adult populations suggest BAT has high specificity and useful sensitivity for distinguishing wheat-allergic individuals (including conventional WDEIA and hydrolyzed wheat protein-induced WDEIA phenotypes).

This review aims to consolidate current knowledge on the diagnostic, clinical, and methodological applications of the basophil activation test (BAT) in IgE-mediated wheat allergy.

## 2. Materials and Methods

A comprehensive literature search was conducted across PubMed, Web of Science, and Scopus to identify original studies assessing the clinical utility of the basophil activation test (BAT) in IgE-mediated wheat allergy. The search included articles published up to May 2025 and was limited to the English language. The following search terms were used in various combinations: (“wheat allergy” OR “wheat-dependent exercise-induced anaphylaxis” OR “WDEIA” OR “hydrolyzed wheat protein allergy” OR “HWP allergy”) AND (“basophil activation test” OR “BAT” OR “CD63” OR “CD203c”). Additional terms included the following: “immediate-type wheat allergy”, “component-resolved diagnosis”, “omalizumab wheat allergy”, “grass pollen cross-reactivity wheat”, “Tri a 14”, and “ω-5 gliadin allergy”.

The review includes pediatric and adult studies, as well as case reports. To enhance methodological transparency, this narrative review was conducted following the general principles of systematic reporting as outlined in the PRISMA guidelines, adapted for narrative synthesis. Eligible studies included clinical trials, observational cohorts, and case reports involving human participants with confirmed IgE-mediated wheat allergy. Exclusion criteria comprised non-IgE-mediated hypersensitivity, animal studies, and in vitro experiments lacking clinical correlation. Data extraction focused on study design, participant characteristics, BAT methodology (allergen source, activation markers, and cut-off criteria), and diagnostic outcomes. Study quality and clinical relevance were evaluated qualitatively, emphasizing reproducibility and the correlation of BAT findings with clinical history or oral food challenge results.

## 3. Clinical Phenotypes and Immunological Basis of Wheat Allergy

IgE-mediated wheat allergy (WA) can affect both children and adults, manifesting in diverse clinical phenotypes including contact urticaria, baker’s asthma (respiratory allergy), wheat-dependent exercise-induced anaphylaxis (WDEIA), and classic food allergy, particularly in children. WA is characterized by reproducible symptoms occurring within two hours of wheat exposure [6] resulting from a complex immunological interplay between allergen-specific IgE and effector cell activation, particularly mast cells and basophils [7].

In children, IgE-mediated food allergy to wheat (FAw) typically arises following gastrointestinal or cutaneous sensitization. Clinically, symptoms range from mild urticaria and angioedema during early food introduction to severe, potentially life-threatening anaphylaxis [8]. Many of these children have a history of atopic dermatitis [9,10] and may react to structurally similar cereal proteins found in barley, rye, and occasionally oats due to prolamin homology [11,12]. Key molecular biomarkers include omega-5 gliadin (Tri a 19), high-molecular-weight glutenin (Tri a 26), low-molecular-weight glutenin (Tri a 36), and alpha-amylase inhibitors (AAIs) (Table 1) [13,14].

In adults, WA is less common but may occur as part of pollen-food syndrome (PFS), particularly in individuals with seasonal allergic rhinitis or respiratory symptoms combined with oral allergy syndrome or anaphylaxis after consuming wheat or related foods [15]. Sensitization in such cases often involves non-specific lipid transfer proteins (nsLTPs) like Tri a 14 (sharing 50% homology with Tri a 14 from durum wheat) or profilins such as Tri a 12 [13,14,16]. False-positive wheat IgE results may also occur due to cross-reactivity within the Pooideae subfamily, especially in clinically tolerant individuals [14].

Baker’s asthma (BA) is the most prevalent occupational wheat allergy worldwide, typically presenting as rhinitis and asthma after the inhalation of wheat flour [13]. Notably, the ingestion of wheat bread is often tolerated, and many affected individuals are not sensitized to grass pollen [17]. Sensitization in BA primarily targets gluten proteins and specific AAIs—particularly Tri a 27 and Tri a 28 (Table 1) [13,18]. Whole-wheat extracts show high sensitivity in testing, while component-resolved diagnostics (CRD) are essential for specificity [13].

Contact urticaria to wheat proteins (CUwps) often results from exposure to hydrolyzed wheat proteins (HWPs) or gluten in cosmetic products. Delayed urticarial reactions on skin contact are reported in adults without other forms of WA. Among occupational food handlers (bakers, cooks, food operators, and waiters, etc.), wheat is the most frequent cause of flour-related allergy [19]. In cosmetic-related industries, immediate skin reaction was described as well, with sIgE positive for LMW-GS, omega 1,2-gliadins, Tritisol powder, and deamidated gluten (but negative for omega 5-gliadins) [20]. Deamidated gluten may even trigger anaphylaxis in patients who tolerate dietary wheat [13,21].

Conventional wheat-dependent exercise-induced anaphylaxis (CO-WDEIA) usually affects adolescents and adults, producing rapid-onset urticaria, angioedema, or anaphylaxis within 1–4 h of wheat ingestion followed by physical activity. Augmenting cofactors such as NSAIDs or alcohol can enhance reactivity, leading to a broader clinical entity known as wheat allergy dependent on augmentation factors (WALDA) [22,23,24]. While hormonal factors, including menstruation, have been hypothesized to modulate WDEIA thresholds in rare instances, supporting evidence is limited to a single case report and remains inconclusive [23]. WDEIA/WALDA should always be taken into consideration in patients with presumable idiopathic anaphylaxis, intolerance to NSAIDs or intermittent/recurrent acute urticaria [25]. The most specific biomarkers are thermostable omega-5 gliadin Tri a 19 (ω5G) and HMW-glutenin [26]. In some challenging ω5G-negative cases, WDEIA may be caused by another heat-stable protein—Tri a 14 (nsLTP), following cutaneous sensitization with HWPs [27] or by cross-reactivity to grass pollen, in scarce occasions [13,28]. ATIs have been proposed to be relevant in WDEIA as well [2].

Delayed-onset WDEIA has been reported in both pediatric and adult populations, with symptoms manifesting several hours post-exercise [16,29]. Data on basophil activation test performance in delayed-onset WDEIA are scarce. Most published cohorts assessed acute-onset phenotypes [25,26], leaving uncertainty about the BAT’s sensitivity in patients with latency exceeding 2 h post-exercise.

A recent study identified a significant association between WDEIA and genetic variants within the HLA-DP region, particularly implicating the HLA-DPB1*02:01:02 allele [25,30]. While this allele is also present at appreciable frequencies in other global populations, including European and Latin American groups, its relevance to other populations remains under investigation.

An epidemic of HWP-induced WDEIA (HWP-WDEIA) occurred in Japan due to facial soaps containing hydrolyzed wheat proteins, resulting in percutaneous or mucosal sensitization and subsequent systemic reactions [31]. Chinuky et al. described this particular WDEIA subtype, which was presumed to be caused by percutaneous or/and rhino conjunctival sensitization from washing their faces with a particular brand of HWP-containing soap. These cases presented with exercise-induced angioedema or anaphylaxis and tested positive for Glupearl 19S (GP19S), a deamidated gluten hydrolysate, capable of cross-reacting to other HWPs [31,32] and surprisingly, no or low levels of ω5G-specific IgE [33]. A similar case was described in Europe [34].

Furuta et al. described a persisting exercise-induced allergic reaction on desensitization (EIARD)**,** that appeared during physical exercise after eating wheat, following desensitization by rush oral immunotherapy (ROIT), in more than two/three of the patients with WA [35]. At more than 5 years after ROIT, half of the patients were still reported as EIARD-positive, suggesting that exercise continues to be a potent cofactor in eliciting allergic reactions, even after desensitization. EIARDs and CO/HWP-WDEIA seem to differ by eliciting allergen components, prognosis, and clinical manifestations, with EIARDs showing more heterogeneous sensitization, a more favorable prognosis under controlled immunotherapy and being overall less severe, although both can be life-threatening [35].

Grass pollen-related wheat allergy (GPWA) is described as an IgE-mediated wheat allergy phenotype occurring in patients with strong grass pollen sensitization but negative in specific IgE to classic wheat allergens (e.g., ω-5 gliadin) [28]. Clinically, GPWA patients present with acute wheat-induced allergic symptoms (e.g., urticaria and anaphylaxis) following wheat ingestion, often without exercise or other cofactors, in marked contrast to WDEIA, which requires exercise or alcohol/NSAIDs. Immunologically, GPWA is characterized by IgE against water-soluble wheat enzymes, peroxidase-1 and β-glucosidase, which are homologous to grass pollen proteins [28].

Both non-Ig-E-mediated and IgE-mediated mechanisms can coexist in celiac disease patients that underwent a wheat elimination diet and may have lost tolerance. Specific antigens from wheat are selectively targeted in celiac disease, whereas seemingly completely different epitopes elicit IgE-mediated food allergies [36]. In patients with celiac disease on a wheat-free diet, new IgE-mediated wheat allergy can develop upon reintroduction, with symptoms that can range from urticaria/angioedema to anaphylaxis [36,37].

## 4. Diagnosis of IgE-Mediated Wheat Allergy

The diagnostic approach to IgE-mediated wheat allergy (WA) begins with a comprehensive clinical history. Essential elements include the chronology of symptoms, tolerance to wheat under other conditions, co-ingestion of additional foods and the potential role of augmenting cofactors such as exercise, non-steroidal anti-inflammatory drugs (NSAIDs), alcohol, or infections. First-line investigations typically involve skin-prick testing (SPT) and the measurement of serum-specific IgE (sIgE). SPT should initially be performed using standardized whole-wheat extracts or native wheat flour, with the latter offering increased sensitivity due to the presence of intact thermolabile and lipophilic allergens [5]. In cases where CO-WDEIA is suspected, SPT with wheat gluten is recommended [38] whereas for HWP-WDEIA, testing should be carried out with the incriminated products or purified hydrolyzed wheat proteins, if available [4].

Serum sIgE directed against whole-wheat extract exhibits high sensitivity across various wheat-related disorders, including food allergy, baker’s asthma, and WDEIA, but lacks specificity. Both serum wheat-sIgE and ω5G-sIgE (ω-5 gliadin specific IgE) prove to be comparably moderate-accuracy diagnostic biomarkers for IgE-mediated wheat allergy, with the slightly higher sensitivity of ω5G-sIgE making it especially useful for identifying challenging cases such as WDEIA and adult-onset wheat allergy [39,40]. To enhance diagnostic resolution, component-resolved diagnostics (CRD) may be employed. For example, IgE to gliadin subfractions (ω-, α-, β-, and γ-gliadins) are relevant in food allergy and WDEIA; IgE to α-amylase/trypsin inhibitors (AAIs), low- and high-molecular-weight glutenins, and Tri a 37 can improve specificity for food allergy and baker’s asthma, while a multiplex of Tri a 27, 28, 29, 32, and 39 appears particularly useful in occupational settings like BA [13,18,24]. From a diagnostic perspective, thresholds established by Sampson et al. further reinforce the utility of sIgE quantification: serum wheat sIgE levels ≥ 100 kU/L are associated with a 100% positive predictive value (PPV) for clinical reactivity, thereby negating the need for an oral food challenge (OFC), while levels < 0.35 kU/L are strongly predictive of tolerance, correlating with a minor risk of reaction during challenge testing [41].

High wheat-specific IgE levels have been associated with a greater risk of severe allergic reactions such as anaphylaxis, particularly in oral food challenge settings [42]. However, even patients with relatively low wheat-sIgE can experience anaphylaxis, indicating that low IgE levels do not guarantee mild reactions [43]. CRD further reveal qualitative differences in IgE sensitization profiles: children with lower wheat-sIgE levels tend to exhibit a broader spectrum of IgE-binding on immunoblots (often including high-molecular-weight glutenin), whereas those with very high sIgE predominantly recognize lower-molecular-weight gliadin fractions (α-, β-, and γ-gliadins) [12,43]. In the pediatric population it is possible to observe persistent elevated sIgE to wheat, but lower than at diagnosis, even when clinical tolerance is obtained [44]. ω5G-sIgE levels measured in patients’ serum were observed to remain persistently elevated in the majority (>85%) of patients with adult-onset wheat allergy (WA), particularly those with wheat-dependent exercise-induced anaphylaxis (WDEIA) [39,40]. In the context of oral immunotherapy, a ≥50% reduction in sIgE levels was significantly associated with the development of sustained unresponsiveness, highlighting a prognostic role in predicting long-term clinical tolerance [45].

CRD have demonstrated considerable utility in distinguishing true wheat allergy from cross-reactive sensitizations, particularly in regions where pollen–food syndromes are prevalent. Due to structural homology, wheat shares cross-reactive epitopes with grass pollens and non-specific lipid transfer proteins (nsLTPs) [13,15], complicating the diagnostic landscape for wheat-related food allergy. In this context, CRD become especially valuable for identifying clinically relevant sensitization patterns. Sensitization to nsLTPs, such as Tri a 14, should be evaluated in patients with suspected wheat-dependent exercise-induced anaphylaxis (WDEIA), especially in the Mediterranean population where LTP sensitization is endemic. Tri a 14 has also emerged as a key allergen in the context of LTP syndrome, particularly in adults presenting with newly acquired food allergies accompanied by respiratory symptoms [18,46,47].

In wheat allergy, IgG4 plays a limited role in diagnosis, but can serve as a meaningful biomarker for monitoring immunotherapy progress. Reliable diagnostic assays, such as the bead-based epitope approach, confirm that epitope-specific IgE, rather than IgG4, is the key discriminator between allergic and tolerant individuals [48]. Conversely, during wheat oral immunotherapy (OIT), treatment consistently induces a rise in wheat- and ω-5-gliadin-specific IgG4 (and IgG1) alongside reductions in IgE reactivity [45].

Oral food challenge (OFC) with wheat-containing products remains the gold standard for diagnosis. In pediatric WA, open challenges are the most practical and may also be used to assess tolerance. For OFC, escalating doses of whole-wheat flour products are administered up to a cumulative dose of at least 1 g of wheat protein, with dose intervals preferably set at 60 min [13,49]. In CO-WDEIA, challenge protocols begin with an exercise-alone test, followed—if negative—by a gluten challenge. If symptoms do not occur, additional cofactors such as aspirin (1 g) or alcohol may be added before re-challenging with gluten the following day [49]. In highly suspicious but ω5G-negative WDEIA cases, higher allergen doses or multiple cofactors may be required to elicit a response (adding ingestion of alcohol and/or exercise before or between gluten doses).

Serum tryptase levels measured 30–120 min after reaction onset and compared to the baseline (taken ≥24 h after resolution) can aid in supporting the diagnosis of anaphylaxis [50].

When conventional in vitro diagnostic tests yield equivocal results, or when food challenges pose unacceptable risk, the basophil activation test (BAT) emerges as a valuable alternative. The BAT provides a functional measure of basophil responsiveness to specific allergens and has demonstrated greater specificity than sIgE in differentiating truly allergic individuals from those who are merely sensitized. This is particularly relevant in patients with grass pollen co-sensitization, low-level IgE, or exposure to atypical allergen sources such as hydrolyzed wheat proteins. In wheat allergy, BAT responses to defined gluten protein types have yielded higher diagnostic performance than IgE alone [12].

## 5. Basophil Activation Test Methodology

The basophil activation test has proven to be a sensitive diagnostic tool, demonstrating high rates of positivity in individuals with food allergies, which may reduce the reliance on more invasive procedures like oral food challenges. Particularly for wheat allergy, this test offers deeper insights into the IgE-mediated mechanisms involved and would allow the allergist to improve patients’ management and diagnosis of wheat allergies, particularly for children or adults with a history of anaphylactic episodes [51].

The basophil activation test is a functional flow-cytometry that measures the activation of the circulating, live basophils stimulated in vitro with specific allergens [52]. This method assesses variables such as possible cross-linkage of the sIgE or, on the contrary, the absence of cross-reactivity between different proteins. Because the BAT interrogates the final effector cell, it bridges the gap between simple sensitization (detected by sIgE) and clinical reactivity, giving it higher specificity than extract-based sIgE in several studies [53,54].

The BAT should be performed using fresh whole blood collected in heparin or EDTA test tubes kept at room temperature (18–25 °C), and ideally performed within 4 h, but similar results were observed even if stored at 4 °C for 24 h [55,56]. A quantity of 4 mL of blood should be sufficient to test controls and two allergens, with an additional 1,5 mL for every other allergen [57]. If using EDTA, it is recommended to firstly process the blood using calcium and magnesium, to allow the activation of the cells [52,58]. Patients should maintain complete avoidance of suspected foods at least 3 to 4 weeks before the test, to avoid a consequent increase in basophil activation in comparison to the baseline [1]. Antihistaminic medication and topical corticosteroids do not affect the test [59], but systemic steroids and ciclosporin may influence the results [56,60].

In the BAT, the allergens used should only be part of water-soluble solutions, preferably standardized preparations, and kept at −20 to −80 °C. Standardized native gluten proteins’ low solubility in aqueous solutions can be addressed by partial hydrolysis with pepsin [61]. Optimal allergen concentration varies by allergen potency and patient sensitivity. It is best to test the dose range to observe the dose–response. Dilutions of the allergens with phosphate-buffered saline (PBS) can range from 10× to 100,000× and should be tested to better assess the basophil response. For many food allergens, final concentrations in the assay on the order of 0.1 ng/mL to 100 ng/mL often bracket the activation range.

In WDEIA, patients’ basophils often react to ω5G in vitro even without exercise, differentiating them from controls. If investigating WDEIA subtypes, test native vs. hydrolyzed wheat extracts. The fractions should be prepared accordingly (e.g., enzymatically hydrolyzed gluten solution) to tailor the BAT for wheat allergy subtypes [62,63].

Anti-IgE antibodies and/or an IgE-independent activator of basophils fMLP (f-Met-Leu-Phen) are used for positive controls, distinguishing samples that are not viable from the non-responders [64]. The assay is inconclusive if patient’s blood reacts only to fMLP; conversely, if the response is limited to anti-IgE and/or allergen stimulation, it supports the validity of the test [57]. For negative controls, PBS and/or KLH are proposed, measuring baseline activation [53].

The basophils make up no more than 2% of all circulating cells and express specific biomarkers while resting, such as CCR3, CD193, CD123, FcεRI (high-affinity receptor for the Fc region of IgE), and CD203c [65]. Using a wide variety of markers helps with a finer selection of the basophils’ population for gating; however, it has been proven that basophil identification could be performed by at least two markers [57,65].

FcεRI/IgE binding is a common activation mechanism for both basophils and mast cells, responsible for immediate responses in food allergies. Although mast cells are activated in vivo by more molecules due to their variety of receptors, they are tissue-based and thus difficult to obtain [66,67].

**Figure 1:**
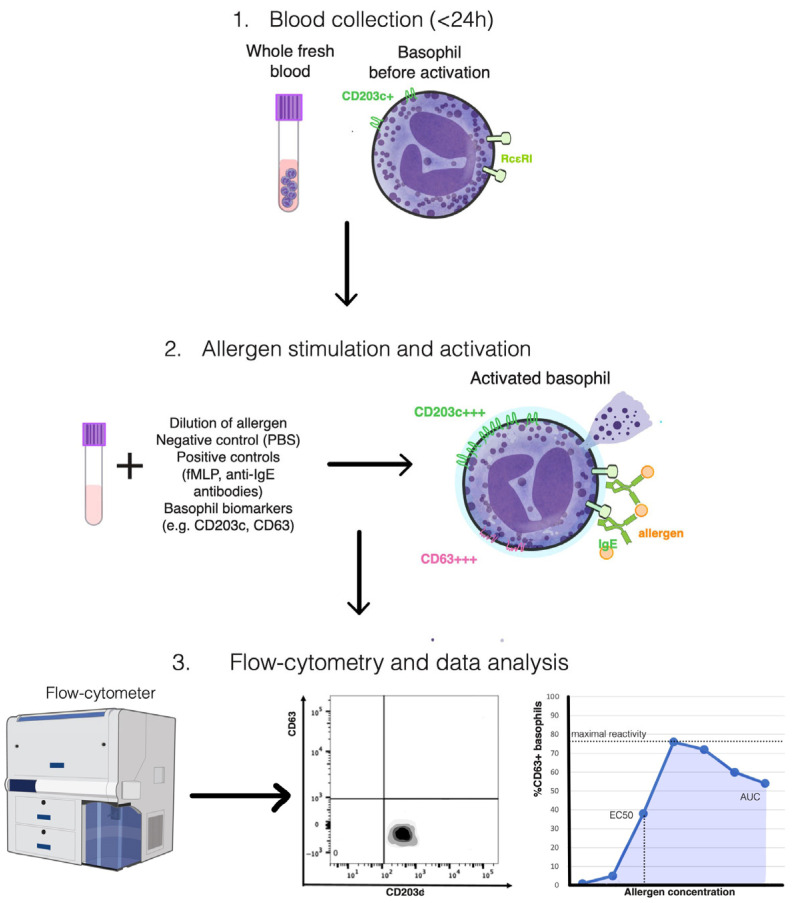
Schematic overview of the basophil activation test (BAT) methodology. Fresh heparinized or EDTA-anticoagulated whole blood (<24 h) containing circulating basophils is first obtained (Step 1). Samples are subsequently incubated with serial dilutions of specific allergens, together with negative (PBS) and positive controls (fMLP and anti-IgE antibodies), to induce in vitro activation (Step 2). During stimulation, the surface expression of activation and identification markers such as CD63 and CD203c is quantified by multiparametric flow cytometry (Step 3). Data analysis enables the characterization of basophil reactivity and sensitivity, expressed through parameters such as maximal reactivity, EC_50_, and the area under the curve (AUC), which collectively reflect the degree of allergen-induced effector cell activation. Illustration created by the authors, conceptually synthesized from multiple published sources and incorporating public-domain elements from the NIH BioArt collection (U.S. National Institutes of Health).

CD123 is expressed on resting basophils as well. It was reported to perform best in different combinations with other markers [68,69,70]; however, it was observed to downregulate during activation in some cases [69,70], leading to possible loss of basophils in the gate. CD203c is expressed on both activated and resting basophils (Figure 1). Its upregulation is related to piecemeal degranulation [71] and increases the possibility of catching as many basophils as possible in the gate. As a result, CD203c is a selection marker useful for phenotyping the basophils [72]. IL-3 enhances CD203c upregulation even without allergens; for clinical BATs, many laboratories therefore omit IL-3 priming to avoid false-positive baselines [73].

CD63 is the main basophil activation marker used [53,57]. It is a lysosomal-associated transmembrane protein, upregulated and exposed on the surface of basophils in IgE-mediated reactions in case of anaphylactic degranulation [74,75]. Its surface exposure is inversely correlated with intracellular concentration of diaminoxidase, which is inversely correlated with histamine release extracellularly [6,76].

The results are referred to as basophil reactivity and sensitivity [77]. The reactivity represents the percentage of gated basophils that expressed the activation markers (CD63-positive) at a given concentration or the concentration at which maximal basophil activation takes place (CD-max) [77,78,79]. Sensitivity is assessed by EC50 (concentration of allergen at which 50% of the maximal basophil response occurs) or CD-sens (inverse of EC50 multiplied by 100), by measurements of basophil reactivity to up to eight allergen concentrations [77,78,79]. The area under the dose–response curve (AUC) is a more recent determination of both reactivity and sensitivity, that takes into account partial anergy during high allergen concentrations and can be measured even during immunotherapy (when the dose–response curve is atypical) [77,78,79].

For a report to be accurate, it is crucial to gate more than 200 basophils. The test requires the inclusion of negative controls to assess spontaneous activation, with the EAACI recommendation of negative threshold of the resting basophils at 2.5%CD63-positive basophils and a positive result at an activation of 5% or a stimulation index (SI) > 2 [53]. Up to 10% of cases are “non-responders”, (no CD63/CD203c on anti-IgE or fMLP), rendering their BAT uninterpretable [12].

As a consequence of high variability between laboratories, it is encouraged to validate cut-offs against oral food challenge outcomes and to report both %CD63^+^ and SI (stimulation index) values in manuscripts to improve comparability [53].

## 6. Clinical Applications of the BAT in Wheat Allergy

The basophil activation test is not a routine diagnostic tool for wheat allergy thus far, but has proved various clinical applications (Table 2): to discriminate between allergic patients and controls, to outline sensitization profiles to distinct wheat allergens (even if they are not commercially available yet), and to correlate more commonly used in vitro tests like specific IgEs that are commercially accessible [13,80].

### 6.1. Diagnostic Utility of BAT in Wheat Allergy

Molecules in wheat, especially gluten and gluten protein types, are proven to induce sturdy dose-dependent basophil activation. Studies employing the basophil activation test have demonstrated that specific fractions, including ω5G and hydrolyzed gluten proteins, provoke the measurable upregulation of activation markers like CD63 and CD203c in sensitized individuals [33,61].

The basophil activation test has emerged as a valuable adjunct for diagnosing IgE-mediated wheat allergy, especially in cases at risk of anaphylaxis where oral food challenges pose significant hazards. In numerous studies (summarized in Table 2), wheat-allergic patients, particularly those with serum IgE to ω5G or positive OFCs to wheat/gluten, show markedly higher basophil activation upon wheat allergen exposure than controls. Tokuda et al. was first to demonstrate that measuring the upregulation of CD203c reliably identified children with immediate-type wheat allergies [82]. Using various wheat extracts and purified ω-5 gliadin (native and recombinant) as stimuli, the BAT achieved a sensitivity of ~85% and specificity of ~77% for native ω5G (corresponding to area under the ROC curve ~0.89, as shown in Table 2), outperforming the conventional wheat-specific IgE test and indicating that the BAT with nω5G is especially sensitive for wheat allergy. In contrast, a higher concentration of recombinant ω5G (rω5G) was required to trigger basophil CD203c upregulation, suggesting a reduced IgE-binding capacity relative to native protein. This discrepancy is likely due to structural and post-translational differences: native allergens retain conformational epitopes and natural modifications (e.g., glycosylation) crucial for IgE recognition, whereas recombinant proteins produced in expression systems may lack these features and thus show diminished immunoreactivity [88,89].

While ω-5 gliadin is a major allergen in wheat-dependent anaphylaxis, other wheat protein fractions can also induce significant basophil activation. BAT studies have shown that water- and salt-soluble extracts (albumins/globulins) and alkali-soluble glutenin fractions from wheat, which contain various non-gliadin allergens, can elicit basophil responses in sensitized patients. For instance, certain non-gluten proteins in the albumin/globulin fraction (such as peroxidase-1 and β-glucosidase) have been implicated in grass pollen-related wheat allergy and trigger basophils in patients who lack ω5G IgE [12,28]. Similarly, HMW-GSs in the glutenin fraction are recognized as important allergens alongside gliadins. Gabler et al. reported that BAT responses to native ω5G and HMW-GSs were the most reliable for identifying WDEIA patients, with higher sensitivity and specificity compared to other gliadin components or low-molecular-weight glutenins [2,61]. These findings support current recommendations to include both ω5G and HMW-GSs in component-resolved IgE testing or BAT panels for WDEIA diagnosis. Moreover, recent BAT experiments confirm that partially hydrolyzed wheat proteins can activate basophils in WDEIA patients, underscoring that allergens beyond intact gluten also contribute to reactivity [2].

In patients with grass pollen-related wheat allergy, the PBS-soluble fraction of wheat proteins elicited the highest basophil activation when peripheral blood basophils were stimulated with various wheat protein extracts [28]. Notably, all six patients exhibited CD203c upregulation in over 10% of basophils in response to the PBS-soluble fraction, suggesting that cross-reactive allergens shared with grass pollens are predominantly water-soluble in nature [28].

### 6.2. Component-Resolved Sensitization Profiles Revealed by the BAT

The allergenic landscape of WDEIA and wheat allergy-related anaphylaxis-like disorders is now recognized to extend beyond ω-5 gliadin. Gabler et al. were the first to systematically explore sensitization profiles beyond ω5G, applying the basophil activation test in a cohort of 12 challenge-confirmed WDEIA patients and 10 non-allergic controls [61]. Using CCR3 for basophil identification and CD63 as the activation marker, their study demonstrated significant reactivity not only to ω5G but also to high-molecular-weight glutenin subunits and whole gluten. Additionally, they established optimal allergen concentrations for dose–response BATs: 4.00 mg/mL for ω-5 gliadins and HMW-GS, 2.00 mg/mL for low molecular weight glutenins (LMW-GS) and gluten, and 0.8 mg/mL for ω-1,2-, α-, and γ-gliadins [61].

Building on this, Faihs et al. evaluated 13 challenge-proven, ω5G-sensitized WDEIA patients and 11 healthy controls. They examined ω-5 gliadin-positive WALDA patients and observed that each had a distinct pattern of basophil reactivity to various wheat allergens beyond ω-5 gliadin [90]. Not only did native gluten and its fractions (gliadins and glutenins) trigger basophil activation, but so did several partially hydrolyzed wheat preparations and albumin/globulin extracts, setting these patients apart from healthy controls. This study also outlined reactivity to HWPs, ATIs, and rye allergens in the same patients by performing the active basophil-release assay, thus identifying sensitization profiles by multiple in vitro cellular testing. Interestingly, over half of the ω-5 gliadin-positive patients also showed basophil activation with rye secalin extract, indicating IgE cross-reactivity between wheat ω-5 gliadin and analogous rye prolamins. Rye γ-70 and γ-35 secalins, as well as barley γ-3 hordein, have been shown to cross-react with ω-5 gliadin due to shared epitope structures, potentially contributing to allergenic responses in sensitized individuals [91]. However, the clinical significance of this finding remains unclear, the study did not evaluate whether those patients could tolerate rye in their diet, and, to date, no published trials have documented clinical reactivity to rye in ω5G-allergic individuals.

Tokuda et al. further emphasized this heterogeneity in pediatric immediate-type wheat allergy by demonstrating basophil activation to multiple wheat fractions [82]. In addition to native gliadins, their study reported significant reactivity to water- and salt-soluble albumins and globulins, as well as alkali-soluble glutenin fractions, reflecting distinct sensitization patterns even in early onset cases.

Insights into the diverse sensitization profiles in WDEIA, particularly to ω5G, have guided experimental strategies for dietary modification. For instance, gluten selectively reduced in ω-5 gliadin content has recently shown to elicit no allergic responses in ω5G-sensitized rat models, supporting the feasibility of tailored hypoallergenic wheat based on dominant sensitization patterns [92,93].

### 6.3. Predictive Value of BAT Parameters for Reaction Severity

None of the BAT parameters so far have been shown to reliably quantify or predict the severity of real-life wheat allergic reactions. All available evidence suggests that while BAT results (e.g., %CD63^+^ activation, CD203c upregulation, CD-sens/EC50, or dose–response AUC) enhance wheat allergy diagnostics and help identify reactive patients, they have not yet provided a proven quantitative measure of reaction severity in wheat allergy. Nilsson et al. studied 24 wheat-allergic children that underwent open oral challenges and observed that basophil sensitivity (e.g., a lower EC50 or higher CD-sens) tended to be greater in those who reacted, although this difference alone was not statistically significant. Combining CD-sens > 150 with wheat-sIgE >20 kUA/L (or ω-5-gliadin > 0.1 kUA/L) correctly identified 83% of positive challenges [83].

Rubio et al. first applied the %CD63^+^/anti-FcεRI ratio to cow’s-milk allergy, showing that a higher ratio discriminated children who remained persistently allergic from those who became tolerant during oral protein tolerance testing [94]. Santos et al. subsequently validated the same index in peanut allergy, where it correlated positively with OFC reaction severity [95]. In wheat allergy, Gabler et al. reported that the ratio was significantly elevated in patients challenged with individual gluten protein types (GPTs) and whole gluten compared with healthy controls; however, no significant association with clinical symptom grades was observed [23,47].

### 6.4. Emerging Wheat Allergens and Novel BAT Stimuli

The exploration of hydrolyzed and fermented wheat extracts as BAT stimuli arises from unmet clinical needs, particularly the diagnostic gap in patients presenting with WDEIA but lacking ω-5-gliadin IgE. These novel preparations aim to capture sensitization profiles missed by conventional serology, expanding the BAT’s role in phenotypes with atypical sensitization mechanisms.

Hydrolyzed wheat proteins (HWPs) constitute a heterogeneous family of wheat-derived allergens that are widely incorporated into cosmetics and processed foods for their potent emulsifying and foaming properties [96,97]. Industrially, HWPs are generated by subjecting gluten to acidic, alkaline, or enzymatic hydrolysis [4]; the choice of hydrolytic conditions produces preparations that differ markedly in molecular-weight distribution, degree of deamidation, and water solubility, each of which can profoundly influence immunogenicity [62]. Partial hydrolysis may expose cryptic epitopes capable of traversing epithelial barriers (skin or gut) and binding to IgEs, whereas extensive hydrolysis can conversely destroy conformational epitopes and reduce allergenicity [62]. Because of these contrasting outcomes, HWPs have become a focal point in recent investigations of WALDA/WDEIA. In the cohort studied by Faihs et al., basophil activation (max %CD63^+^) to both partially hydrolyzed (sHWP) and extensively hydrolyzed (eHWP) extracts showed a strong positive correlation with patients’ serum ω5G-specific IgE, underscoring the clinical relevance of HWP reactivity [87].

In a follow-up study, Gabler et al. tested 12 WDEIA patients via the BAT with various wheat preparations: native gluten, gluten with reduced ω5-gliadins, and partially vs. extensively HWPs [2]. All test solutions triggered significantly higher basophil activation in WDEIA patients compared to controls. The BAT with sHWP was especially effective, yielding 100% sensitivity and 83% specificity at an optimal CD63% cut-off. This suggests that even non-gluten wheat proteins (e.g., albumins such as α-amylase/trypsin inhibitors, which were abundant in the test solutions) can act as cofactor-dependent allergens. The authors conclude that BAT is a useful diagnostic tool for WDEIA, and that previously under-recognized non-gluten proteins (e.g., ATIs) may play a significant role in WDEIA pathogenesis [2].

Alcohol-free wheat beer (afWB), a readily accessible and food-grade extract, was utilized as a test stimulus in the basophil activation test by Faihs et al. in patients with ω5G-positive WDEIA/WALDA [87]. The afWB induced strong basophil activation comparable to purified gluten protein types, and it also elicited positive skin-prick test responses in the majority of patients while remaining negative in most healthy controls [87].

The Ogino et al. study identified a unique phenotype of wheat allergy in adults who had negative ω5G IgE but high grass pollen IgE, termed grass pollen-related wheat allergy [28]. Six such patients (some with exercise-induced reactions) were compared to 17 grass-pollen-allergic controls and 29 patients with other wheat allergies (18 conventional WDEIA and 11 HWP-WDEIA). A BAT with fractionated wheat proteins showed that all six GPWA patients’ basophils reacted to water-soluble wheat proteins (albumin/globulin fraction), whereas their responses to gluten fractions were absent or minimal. Using immune-blot and mass spectrometry, two water-soluble wheat allergens, peroxidase-1 and β-glucosidase, were identified as the IgE targets. In patient sera, IgE binding to these proteins was inhibited by pre-incubation with grass pollen extract, confirming IgE cross-reactivity between grass pollen and these wheat proteins. Newly developed specific IgE assays for peroxidase-1 and β-glucosidase were positive in three and four of six GPWA patients, respectively, but in only 2 of 29 other wheat-allergy controls [28].

Thus, novel stimuli such as HWP and afWB extend the BAT’s reach to non-classical wheat allergy phenotypes, providing a practical approach where standard IgE testing fails to explain clinical reactivity.

### 6.5. BAT as a Biomarker for Biologic and Immunotherapy Monitoring

The use of biologics targeting Th2-driven inflammation is being increasingly explored as either standalone therapy or as an adjunct to immunotherapy for food allergies, particularly to reduce side effects and improve the feasibility of IT in highly reactive patients. In such individuals, elevated basophil activation has emerged as a valuable biomarker for identifying those who may benefit from omalizumab—either during oral immunotherapy (OIT) or through a tailored treatment schedule aimed at minimizing adverse effects. Interestingly, omalizumab produces two opposing effects on basophils: it reduces FcεRI receptor density while simultaneously enhancing their intrinsic sensitivity to allergens [98,99]. Both effects can be tracked using the BAT and have been shown to correlate with clinical outcomes.

Two studies by Chinuki et al. have explored the role of the BAT in monitoring therapeutic response to omalizumab in patients with wheat-dependent exercise-induced anaphylaxis, with emphasis on both the short- and long-term treatment effects.

The 2020 pilot study focused on patients with hydrolyzed wheat protein-induced WDEIA (HWP-WDEIA) and evaluated the effect of short-term omalizumab (150 mg every 4 weeks for three doses) [100]. This study demonstrated a transient reduction in CD203c expression-based BAT responses to wheat fractions (HWP, PBS, EtOH, and Alkali). Significant suppression was observed at 4 weeks post-treatment initiation, but basophil activation rates returned to baseline levels by week 12. Notably, the study did not include food challenge testing, and thus correlations between BAT suppression and symptom resolution could not be evaluated.

The 2023 study conducted a longer-term, multicenter phase 2 trial with individualized omalizumab dosing (150–600 mg) over 44 weeks, followed by a 24-week observation period [85]. The cohort included both HWP-WDEIA and CO-WDEIA patients. BAT suppression below the clinically significant threshold of 10% was achieved in over 80% of patients during treatment, and this immunologic improvement was paralleled by clinical benefit, with 68.8% of patients remaining symptom-free upon wheat reintroduction. However, BAT suppression and clinical tolerance diminished progressively after cessation of omalizumab, indicating that the desensitization achieved was not durable without continued therapy. This study supports the BAT as a biomarker not only for immunologic sensitization but also for guiding dietary reintroduction and risk assessment in clinical practice [85].

Together, these studies reinforce the utility of the BAT as a dynamic, treatment-dependent tool to evaluate omalizumab efficacy in wheat allergy. The comparison highlights that longer duration and personalized dosing of omalizumab are more effective in achieving and maintaining BAT suppression, and that the BAT correlates with clinical outcomes when used in a longitudinal, integrative approach. Limitations common to both studies include the lack of randomized placebo control and the absence of standardized oral food challenges during treatment, which should be addressed in future research to validate the BAT’s predictive value for clinical desensitization.

### 6.6. Guiding Dietary Reintroduction with BAT Suppression Thresholds

The methodological advances described above form the basis for translating the BAT from a research tool into a clinically meaningful test. At this point, it becomes essential to consider how these technical developments align with patient-centered outcomes. By linking laboratory activation profiles to real-world clinical scenarios, the BAT also demonstrated its potential as a valuable instrument for guiding therapeutic decisions.

In the 2023 study, Chinuki et al. described BAT suppression as a basophil activation rate below 10% across all tested wheat fractions and used it as a clinical threshold to guide the lifting of wheat elimination diets. Patients who met this immunologic criterion were permitted to resume regular wheat consumption under medical supervision. Notably, 68.8% of these patients (11 of 16) remained entirely symptom-free upon reintroduction, and the incidence of allergic reactions remained below 15% during the omalizumab treatment phase [100]. This underscores the clinical validity of the BAT as a surrogate marker for temporary tolerance to wheat. However, after omalizumab discontinuation, both basophil reactivity and clinical symptoms gradually recurred, with the proportion of patients experiencing allergic reactions increasing to 46.7% by week 60, suggesting a loss of desensitization. These findings imply that the BAT can predict short-term clinical tolerance.

While omalizumab has shown promise in facilitating desensitization in IgE-mediated food allergies, such as in the MacGinnitie et al. study [10], where patients with peanut allergies maintained high levels of tolerance after discontinuing omalizumab, this outcome does not appear to extend to WDEIA. As the authors emphasized, long-term omalizumab therapy combined with routine wheat ingestion is not sufficient to induce lasting desensitization in adult patients with wheat allergy and the BAT can be a biomarker for decisions regarding dietary reintroduction [85].

## 7. Limitations

Logistical constraints: Fresh whole blood is mandatory as basophil reactivity rapidly declines within hours of venipuncture. Current best practice recommends sample processing within 4 h, making same-day collection, on-site flow cytometry, and trained personnel non-negotiable prerequisites. Attempts to ship samples or use cryopreserved cells markedly reduce assay performance. Furthermore, specialized infrastructure is essential—a three-laser flow cytometer, allergen-stimulation hood, and operators familiar with complex gating are required—limiting BAT implementation to tertiary centers and specialized laboratories [53].

Biological limitations: The “non-responder” phenomenon remains a major challenge, rendering a subset of assays uninterpretable and necessitating repeat venipuncture or alternative diagnostic methods. Cohorts in wheat-dependent exercise-induced anaphylaxis (WDEIA) are generally small (<50 patients), reflecting the rarity of the condition. Published evidence on pediatric applications of the BAT in wheat allergy remains extremely limited, with only two dedicated studies identified among over 30 BAT-related publications. This scarcity restricts the extrapolation of adult-derived cut-off values and hampers the development of age-specific reference ranges. By contrast, robust evidence exists for the BAT in sesame and peanut allergy, characterized by larger, more homogeneous datasets with consistently high diagnostic accuracy in IgE-mediated food allergy [39]. Additionally, the BAT has demonstrated utility in monitoring baked and cooked egg and milk tolerance in children, reducing the need for oral food challenges [74,75] and in correlating basophil reactivity with severity during peanut oral food challenges [101].

Assay-standardization issues: Allergen preparation remains highly heterogeneous across studies. Most WDEIA protocols rely on labor-intensive gluten protein fractions that require sequential salt/ethanol extraction, dialysis, and lyophilization to achieve water solubility. Recombinant or highly purified targets such as MM1 gliadin or peroxidase-1 have shown promise, but their production is costly and not yet commercially available. Consequently, inter-laboratory reproducibility remains poor, and no universally accepted allergen panel has been established. Additionally, post-translational modifications (e.g., deamidation and glycosylation) and chemical interactions affecting protein conformation can modulate folding and allergenicity, further complicating standardization across preparations [102,103]. Cut-off thresholds also vary widely (e.g., 5% vs. 15% CD63^+^ basophils), further complicating interpretation or flipping a result from positive to negative [28,53].

Evidence-based gaps: The current literature is dominated by underpowered pediatric series and single-center adult studies, with no multicenter validation trials using harmonized standard operating procedures. Longitudinal data evaluating the BAT as a biomarker for immunotherapy monitoring or tolerance prediction are virtually absent, except for one omalizumab-treated cohort. Finally, formal cost-effectiveness and reimbursement analyses remain unavailable, limiting clinical adoption and payer acceptance [65].

## 8. Conclusions

The BAT surpasses routine allergy tests when component allergens are used. Using ω-5 gliadin and HMW-glutenin, the BAT routinely delivers ≥ 85% sensitivity and ≥77% specificity, matching or exceeding SPT and sIgE while dramatically reducing the need for risky food challenges.

The dual-component BAT (ω-5 gliadin + HMW-glutenin) is definitive for WDEIA. Combining these two markers identifies virtually every WDEIA case with minimal false positives, making BAT the tool of choice when diagnosis is uncertain.

Personalized panels reveal hidden cross-reactivity. The BAT exposes unique reactivity patterns to ATIs, rye secalins, hydrolyzed wheat proteins, and other cofactors, information essential for precise diet advice and tailored immunotherapy.

Component-resolved data informs hypoallergenic wheat breeding. Pinpointing immunodominant proteins provides a rational target list for gene-editing or breeding “designer grains” that retain baking quality but shed allergenicity.

The BAT is a rising biomarker for therapy monitoring. In omalizumab studies, <10% basophil activation correlates with clinical protection, suggesting that the BAT could replace provocation challenges as an outcome read-out in future trials.

Standardization is the critical next step. Widespread adoption hinges on harmonized allergens, gating strategies, and external quality controls—an international effort paralleling sIgE assay standardization.

## Figures and Tables

**Table 1:** Wheat allergens and their clinical relevance across IgE-mediated phenotypes.

Allergen (Tri a)	Protein Family/Type	Associated Phenotype(s)	Sensitization Route	Key Notes/ Diagnostic Relevance
**Tri a 19 (ω-5 gliadin)**	Prolamin	Classic WA, WDEIA, WALDA	Oral	Major marker for WDEIA; thermostable; key BAT; and sIgE target.
**Tri a 14 (nsLTP)**	Non-specific lipid transfer protein	WDEIA (HWP-negative cases), Pollen-food syndrome	Cutaneous/Oral	Cross-reactivity with peach, grass pollen; relevant in Asia.
**Tri a 12 (Profilin)**	Profilin	Pollen-food syndrome	Respiratory	Causes mild oral symptoms; cross-reactive with grass pollen.
**Tri a 27/Tri a 28**	α-Amylase/Trypsin inhibitors (ATI)	Baker’s asthma, WDEIA (minor role)	Inhalation/Oral	Occupational exposure; enhances innate immune response.
**Tri a 26 (HMW-GS)**	Glutenin (high molecular weight)	WDEIA, FAw	Oral	Marker of severe phenotypes; thermostable.
**Tri a 36 (LMW-GS)**	Glutenin (low molecular weight)	FAw, HWP contact urticaria	Oral/Cutaneous	Found in cosmetic-induced sensitization.
**Peroxidase-1**	Enzyme	GPWA	Respiratory/Oral	Homologous to grass pollen peroxidase; diagnostic confusion possible.
**Tri a 37 (β-glucosidase)**	Enzyme	GPWA	Respiratory/Oral	Cross-reactivity with grass pollen β-glucosidase.
**Deamidated gluten**	Modified gluten hydrolysate	HWP-WDEIA, contact urticaria	Cutaneous	Triggers reactions even in dietary wheat tolerance.
**ω-1,2 gliadin**	Gliadin fraction	Cosmetic-related allergy	Cutaneous	Detected in HWP reactions; cross-reacts with deamidated peptides.

Abbreviations: ATI, α-amylase/trypsin inhibitor; BA, baker’s asthma; BAT, basophil activation test; FAw, food allergy to wheat; GPWA, grass pollen-related wheat allergy; HMW-GSs, high-molecular-weight glutenin subunits; HWP, hydrolyzed wheat protein; LMW-GS, low-molecular-weight glutenin subunits; nsLTP, non-specific lipid transfer protein; sIgE, specific immunoglobulin E; WALDA, wheat allergy dependent on augmentation factors; WA, wheat allergy; WDEIA, wheat-dependent exercise-induced anaphylaxis.

**Table 2:** Basophil activation test in IgE-mediated wheat allergy and WDEIA: summary of key studies.

Year	First Author	Study Type	Population	Highlights	Allergens Tested	Markers	Allergen Concentrations Used
2005	Ehrlich R. et al. [81]	Case report: Occupational wheat allergy/Baker’s asthma	One adult male baker with asthma	Stronger reactivity to rye vs. wheat (37% vs. 17% CD63^+^ basophils); confirmed by bronchial challenge	Purified rye and wheat flour extracts	CD63	Bühlmann extract
2009	Tokuda R et al. [82]	Pediatric BAT study	32 wheat-allergic children vs. 27 tolerant controls	85% sensitivity, 77% specificity; AUC 0.89; better than sIgE	PBS, EtOH, Alkali, purified native ω5G, recombinant ω5G	CD203c	0.01–0.1–1–10 µg/mL
2012	Chinuki Y et al. [33]	Distinct WDEIA phenotypes	10 WDEIA patients (5 CO-WDEIA, 5 HWP-WDEIA)	Differentiated CO-WDEIA vs. HWP-WDEIA	HWP, ω5G	CD203c	0.0001–1 µg/mL
2013	Nilsson N et al. [83]	Pediatric BAT study	24 wheat-allergic children undergoing oral challenge	Wheat CD-sens > 150 and wheat IgE > 20 kUA/L/ω-5 gliadin IgE > 0.1 kUA/L	Wheat extract, recombinant ω-5 gliadin, HWP, timothy grass	CD203c, CD63	Serial dilutions used to derive CD-sens;
2018	Zhang Q [84]	Abstract (JACI)	Patients with WDEIA	Higher %CD63^+^ basophils in WDEIA vs. controls	Crude wheat extract	CD63	Not specified
2021	Gabler AM et al. [61]	Component-specific BAT (GPT-BAT)	12 challenge-confirmed WDEIA adults vs. 10 healthy controls	ω5G AUC 0.91; 100% sensitivity and HMW-glutenin: 70–100% specificity	ω5G, ⍵ 1,2-gliadins, ⍺- and Ɣ-gliadins, HMG-GS, LMW-GS, gluten	CCR3, CD63	0.08–4.0 mg/mL
2022	Gabler AM et al. [2]	Gluten vs. HWP in WDEIA	13 WDEIA-patients vs. 13 exercise-tolerant controls	sHWP BAT 100% sens., 83% spec.; α-ATIs implicated	saline extracts of gluten, gluten with reduced content of ω5G, sHWP, eHWP	CCR3, CD63	2.10 mg/mL gluten, 2.05 mg/mL gluten with reduced content of ω5G, 3.96 mg/mL sHWP, 3 mg/mL eHWP
2023	Chinuki Y et al. [85]	Omalizumab treatment study	20 WDEIA adults (omalizumab trial, no controls)	82% Achieved BAT < 10% during treatment	HWP, PBS, EtOH, Alkali, purified ω5G	CD203c, IgE	HWP: 0.1/1 µg/mL, PBS: 1/10 µg/mL, EtOH: 1/10 µg/mL, Alkali: 1/10 µg/mL, purified ω5G: 0.1/1 µg/mL
2023	Aoki Y et al. [86]	Novel gliadin epitope (MM1) identification	42 WDEIA patients, 8 non-WDEIA wheat-allergic adults, 20 healthy controls	MM1-activated basophils in all 14 tested WDEIA; none in non-allergic	r α/β gliadin MM1	CD123^+^/HLA-DR^−^, CD203c	Not specified
2024	Faihs V et al. [87]	Sensitization profiling in WALDA	13 WDEIA/WALDA adults vs. 11 healthy controls	BAT activated across all tested allergens; individualized profiles	gluten, HMW-GS, ATIs, afWB, extensive HWPs, slightly HWPs, rye gluten, and secalin	CD123^+^/HLA-DR^−^, CD203c	Wheat, rye, gluten, HMW-GS: 4000/2000/800 µg/mL, ATIs: 400/200/80/40 µg/mL, afWB:1:10/1:100, e&pHWP: 1:5/1:10/1:50, rye secalins: 800 µg/mL

Abbreviations: afWB, alcohol-free wheat beer; AIT, allergen immunotherapy; Alkali, alkali extraction fraction of wheat protein; AUC, area under the curve; ATIs, α-amylase/trypsin inhibitors; BAT, basophil activation test; beta-G, beta-glucosidase; CRD, component-resolved diagnostics; eHWPs, extensively treated hydrolyzed wheat proteins; EtOH, ethanol extraction fraction of wheat protein; GP, grass pollen; HMW-GSs, high-molecular-weight glutenin subunits; HWP, hydrolyzed wheat protein; HWPs, hydrolyzed wheat proteins; LMW-GS, low-molecular-weight glutenin subunits; NPV, negative predictive value; OIT, oral immunotherapy; PBS, phosphate-buffered saline soluble fraction of wheat protein; PPV, positive predictive value; px-1, peroxidase-1; r α/β-gliadin, recombinant alpha/beta gliadin; ROC, receiver operating characteristic; sHWPs, slightly treated hydrolyzed wheat proteins; sIgE, specific immunoglobulin E; SI, stimulation index; WDEIA, wheat-dependent exercise-induced anaphylaxis; ω5G, omega-5 gliadin.
